# Bis(4-amino­pyridinium) tetra­iodido­cad­mate monohydrate

**DOI:** 10.1107/S1600536812034447

**Published:** 2012-08-08

**Authors:** Qiaozhen Sun, Songyi Liao, Junjun Yao, Junke Wang, Qiongjiali Fang

**Affiliations:** a Department of Materials Chemistry, School of Materials Science and Engineering, Key Laboratory of Nonferrous Metal of Ministry of Education, Central South University, Changsha 410083, People’s Republic of China

## Abstract

The title compound, (C_5_H_7_N_2_)_2_[CdI_4_]·H_2_O, contains one [CdI_4_]^2−^ anion, two prontonated 4-amino­pyridine mol­ecules and one water mol­ecule in the asymmetric unit. In the anion, the Cd^II^ atom is coordinated by four I atoms in a slightly distorted tetra­hedral geometry. The [CdI_4_]^2−^ anion and the water mol­ecule are bis­ected by a crystallographic mirror plane perpendicular to the *c*-axis direction, with the Cd^II^ atom, two of the I atoms and the atoms of the water mol­ecule located on this plane. The crystal packing is stabilized by inter­molecular N—H⋯I, N—H⋯O and O—H⋯I hydrogen bonds and by π–π stacking inter­actions [centroid–centroid distance = 3.798 (3) Å) between pyridine rings, which build up a three-dimensional network.

## Related literature

For background literature on the magnetism, anti­viral activity and luminescence of organic–inorganic hybrid compounds, see: Bauer *et al.* (2003 ▶); Cavicchioli *et al.* (2010 ▶); Li *et al.* (2007 ▶). For ion channel inhibitor properties of 4-amino­pyridine, see: Picolo *et al.* (2003 ▶). For metal complexes of 4-amino­pyridine, see: Das *et al.* (2010 ▶); Ivanova *et al.* (2005 ▶); Jebas *et al.* (2009 ▶); Kulicka *et al.* (2006 ▶); Rademeyer *et al.* (2007 ▶); Zaouali Zgolli *et al.* (2009 ▶). For bond-length data, see: Anderson *et al.* (2005 ▶); Hines *et al.* (2006 ▶).
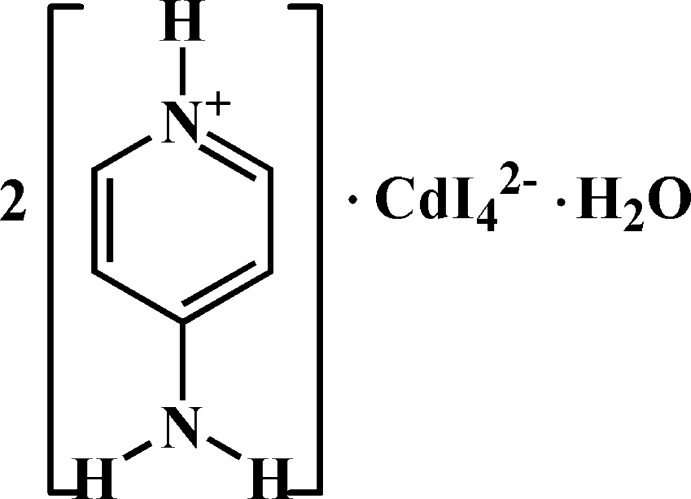


## Experimental

### 

#### Crystal data

(C_5_H_7_N_2_)_2_[CdI_4_]·H_2_O
*M* *_r_* = 828.27
Orthorhombic, 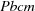

*a* = 7.3987 (2) Å
*b* = 14.7348 (4) Å
*c* = 18.7286 (4) Å
*V* = 2041.76 (9) Å^3^
*Z* = 4
Mo *K*α radiation
μ = 7.12 mm^−1^
*T* = 293 K
0.40 × 0.24 × 0.20 mm

#### Data collection

Bruker SMART CCD diffractometer
Absorption correction: multi-scan (*SADABS*; Sheldrick, 1996 ▶) *T*_min_ = 0.145, *T*_max_ = 0.340
11964 measured reflections
1860 independent reflections
1755 reflections with *I* > 2σ(*I*)
*R*_int_ = 0.052

#### Refinement

*R*[*F* ^2^ > 2σ(*F* ^2^)] = 0.025
*wR*(*F* ^2^) = 0.064
*S* = 1.02
1860 reflections
102 parameters
3 restraints
H atoms treated by a mixture of independent and constrained refinement
Δρ_max_ = 0.91 e Å^−3^
Δρ_min_ = −0.72 e Å^−3^

### 

Data collection: *SMART* (Bruker, 2000 ▶); cell refinement: *SAINT* (Bruker, 2000 ▶); data reduction: *SAINT*; program(s) used to solve structure: *SHELXTL* (Sheldrick, 2008 ▶); program(s) used to refine structure: *SHELXTL*; molecular graphics: *SHELXTL*; software used to prepare material for publication: *SHELXTL*.

## Supplementary Material

Crystal structure: contains datablock(s) global. DOI: 10.1107/S1600536812034447/zl2495sup1.cif

Additional supplementary materials:  crystallographic information; 3D view; checkCIF report

## Figures and Tables

**Table 1:** Hydrogen-bond geometry (Å, °)

*D*—H⋯*A*	*D*—H	H⋯*A*	*D*⋯*A*	*D*—H⋯*A*
N1—H1*B*⋯O*W*1^i^	0.86	2.03	2.886 (5)	173
N2—H2*A*⋯I2^ii^	0.86	3.12	3.938 (4)	161
N2—H2*B*⋯I2	0.86	3.04	3.843 (4)	157
O*W*1—H*W*1*A*⋯I2^ii^	0.83 (2)	3.24 (1)	3.828 (4)	130 (1)
O*W*1—H*W*1*A*⋯I2^iii^	0.83 (2)	3.24 (1)	3.828 (4)	130 (1)
O*W*1—H*W*1*A*⋯I1	0.83 (2)	3.27 (4)	3.704 (5)	116 (3)
O*W*1—H*W*1*B*⋯I3^iv^	0.85 (2)	2.99 (2)	3.761 (5)	152 (4)
O*W*1—H*W*1*A*⋯I1	0.83 (2)	3.27 (4)	3.704 (5)	116 (3)

Symmetry codes: (i) 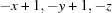; (ii) 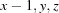; (iii) 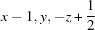; (iv) 
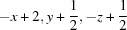
.

**Fig. 1.**
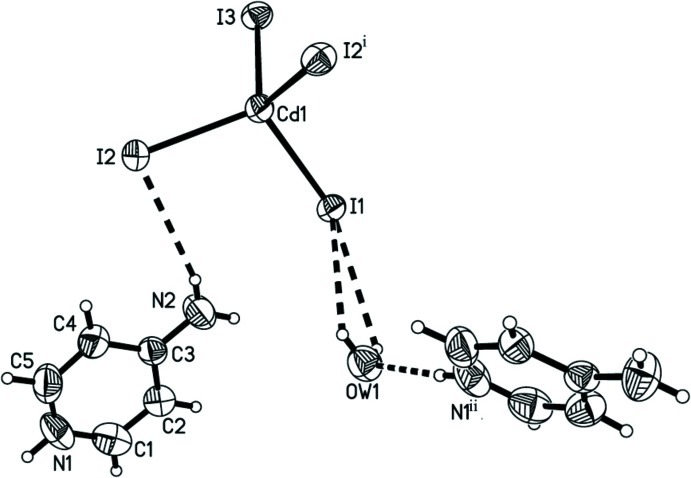
The title compound showing the atom-numbering scheme, with displacement ellipsoids shown at the 50% probability level; hydrogen atoms are drawn as spheres of arbitrary radius. Hydrogen bonds are shown as dashed lines. [Symmetry codes: (i) x, y, -z+0.5; (ii) -x+1, -y+1, 0.5+z]

**Fig. 2.**
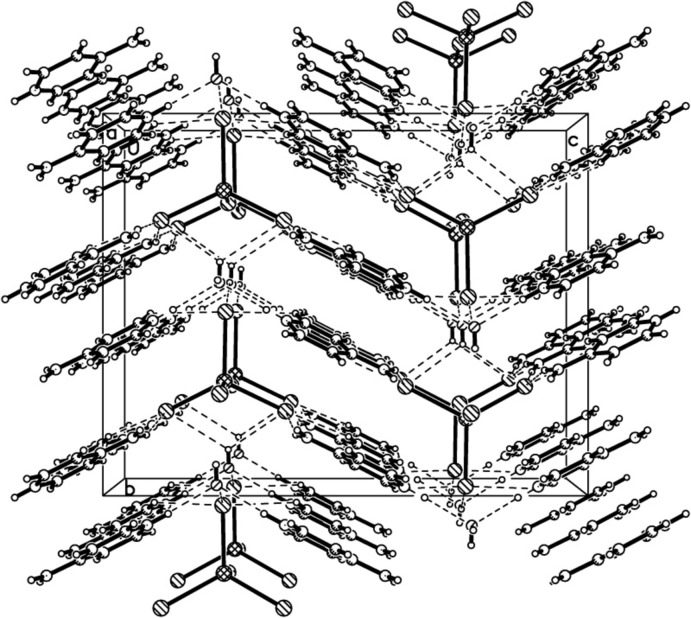
A packing diagram of the title compound, viewed in perspective along the a axis.

**Fig. 3.**
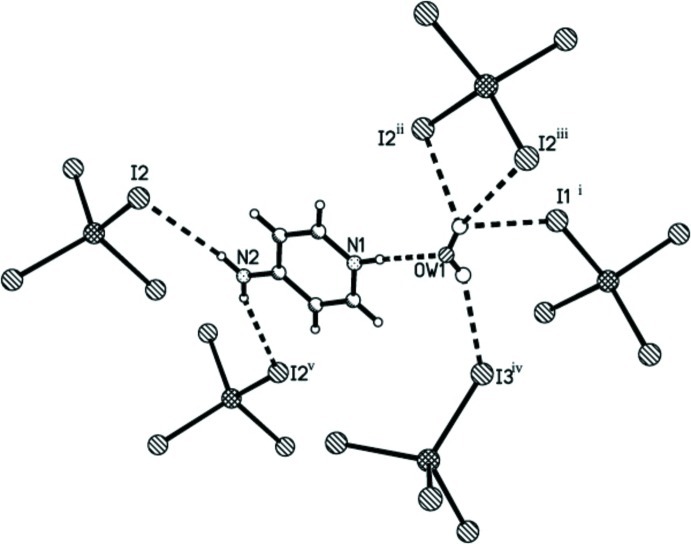
A view of the various N—H···I, N—H···O and O—H···I hydrogen bonds in the (1 1 2) plane, with hydrogen bonds shown as dashed lines. [Symmetry codes: (i) -x+1, -y+1, -z; (ii) -x+2, -y+1, -z; (iii) -x+2, -y+1, z-0.5; (iv) x-1, -y+0.5, z-0.5; (v) x-1, y, z.]

## supplementary crystallographic information

### Comment

Studies of hydrogen bonds connecting organic-inorganic hybrid compounds continue to be a topic of intense research in crystal engineering because such compounds not only allow for rational bottom-up construction but hydrogen bonds also effectively regulate the molecular architecture. Hydrogen bond connected organic-inorganic hybrid compounds can exhibit novel properties related to e.g magnetism, luminescence, antiviral activity and even multifunctional properties (Bauer *et al.*, 2003; Cavicchioli *et al.*, 2010; Li *et al.*, 2007). The protonated form of 4-aminopyridine (4-AP) has hydrogen-bonding capability at both ends of the molecule, and it is also biologically active and can be used as a K^+^ and Ca^2+^ channel inhibitor (Picolo *et al.*, 2003). Structures of 4-AP with the metals Mn^II^, Co^II^, Cu^II^, Ni^II^, Sn^IV^, Sb^V^ and Pd^II^ have been reported (Das *et al.*, 2010; Ivanova *et al.*, 2005; Jebas *et al.*, 2009; Kulicka *et al.*, 2006; Rademeyer *et al.*, 2007; Zaouali Zgolli *et al.*, 2009). Here we report the crystal structure of the title compound, which is a salt that comprises two symmetry related 4-AP cations and a complex [CdI_4_]^2-^ anion, Fig. 1. The [CdI_4_]^2-^ anion and the water molecule are bisected by a crystallographic mirror plane perpendicular to the c-axis direction, with the the atoms Cd1, I1, I3 and the water molecule located on this plane at *x*, *y*, 1/4. In the anion, the Cd^II^ ion is coordinated by four I atoms, exhibiting a slightly distorted tetrahedral geometry. The mean Cd···I bond distance is 2.78 Å, which is similar to that of related compounds reported in the literature (Hines *et al.*, 2006).

In the cation, the nitrogen atom of the pyridine ring is protonated. Both of the nitrogen atoms of 4-AP are not metal coordinated, but are instead involved in an extensive hydrogen bonding network that includes the amine hydrogen atoms and the iodine atoms, the protonated pyridyl hydrogen atom, and the water molecule. The bond distances and bond angles of the 4-AP cation are comparable with values reported earlier for its uncomplexed form (Anderson *et al.*, 2005).

Packing of the title complex (Fig. 2 and Fig. 3) is facilitated through π–π interactions between pyridine rings [ring centroid distance: 3.798 (3) Å], through the N—H···I hydrogen bonds between the [CdI_4_]^2-^ anions and the 4-AP cations, and through O—H···I and N—H···O hydrogen bonds, which link the components of the structure into a three dimensional network.

### Experimental

A mixture of CdI_2_ (0.36 g, 0.98 mmol), pyridine-2,3-dicarboxylic acid (0.08 g, 0.48 mmol), and 4-aminopyridine (0.06 g, 0.64 mmol) in H_2_O (12.0 mL) was sealed in a 20 mL stainless-steal reactor with Teflon liner and heated at 423 K for 60 h under autogenous pressure. Colorless block crystals were collected after the reaction solution was cooled. Yield: 16%. IR: 3314(*s*), 1653(*s*), 1608(*s*), 1526(*s*), 1407(*s*), 1197(*m*), 993(*m*), 865(w), 806(*m*), 758(*m*), 715(w), 495(*m*).

### Refinement

All of the non-hydrogen atoms were refined with anisotropic thermal displacement parameters. The O—H distances of water molecules were restrained to 0.84 Å with a standard deviation of 0.001 Å. The other H atoms were not located in the difference map and placed in calculated positions using the riding model approximation with C—H distances of 0.93 Å and an N—H distances of 0.86 Å. U_iso_(H) were set to 1.2U_eq_(C, N) or 1.5U_eq_(O).

### Crystal data

**Table:**

(C_5_H_7_N_2_)_2_[CdI_4_]·H_2_O	*F*(000) = 1488
*M**_r_* = 828.27	*D*_x_ = 2.694 Mg m^−^^3^
Orthorhombic, *P**b**c**m*	Mo *K*α radiation, λ = 0.71073 Å
Hall symbol: -P2c2b	Cell parameters from 7689 reflections
*a* = 7.3987 (2) Å	θ = 2.6–28.2°
*b* = 14.7348 (4) Å	µ = 7.12 mm^−^^1^
*c* = 18.7286 (4) Å	*T* = 293 K
*V* = 2041.76 (9) Å^3^	Block, colourless
*Z* = 4	0.40 × 0.24 × 0.20 mm

### Data collection

**Table:**

Bruker SMART CCD diffractometer	1860 independent reflections
Radiation source: fine-focus sealed tube	1755 reflections with *I* > 2σ(*I*)
Graphite monochromator	*R*_int_ = 0.052
φ and ω scans	θ_max_ = 25.0°, θ_min_ = 2.8°
Absorption correction: multi-scan (*SADABS*; Sheldrick, 1996)	*h* = −8→8
*T*_min_ = 0.145, *T*_max_ = 0.340	*k* = −17→14
11964 measured reflections	*l* = −22→22

### Refinement

**Table:**

Refinement on *F*^2^	Secondary atom site location: difference Fourier map
Least-squares matrix: full	Hydrogen site location: inferred from neighbouring sites
*R*[*F*^2^ > 2σ(*F*^2^)] = 0.025	H atoms treated by a mixture of independent and constrained refinement
*wR*(*F*^2^) = 0.064	*w* = 1/[σ^2^(*F*_o_^2^) + (0.0406*P*)^2^ + 1.5671*P*] where *P* = (*F*_o_^2^ + 2*F*_c_^2^)/3
*S* = 1.02	(Δ/σ)_max_ = 0.001
1860 reflections	Δρ_max_ = 0.91 e Å^−^^3^
102 parameters	Δρ_min_ = −0.72 e Å^−^^3^
3 restraints	Extinction correction: *SHELXTL* (Sheldrick, 2008), Fc^*^=kFc[1+0.001xFc^2^λ^3^/sin(2θ)]^-1/4^
Primary atom site location: structure-invariant direct methods	Extinction coefficient: 0.00433 (17)

### Special details

**Table:**

Geometry. All esds (except the esd in the dihedral angle between two l.s. planes) are estimated using the full covariance matrix. The cell esds are taken into account individually in the estimation of esds in distances, angles and torsion angles; correlations between esds in cell parameters are only used when they are defined by crystal symmetry. An approximate (isotropic) treatment of cell esds is used for estimating esds involving l.s. planes.
Refinement. Refinement of F^2^ against ALL reflections. The weighted R-factor wR and goodness of fit S are based on F^2^, conventional R-factors R are based on F, with F set to zero for negative F^2^. The threshold expression of F^2^ > 2sigma(F^2^) is used only for calculating R-factors(gt) etc. and is not relevant to the choice of reflections for refinement. R-factors based on F^2^ are statistically about twice as large as those based on F, and R- factors based on ALL data will be even larger.

### Fractional atomic coordinates and isotropic or equivalent isotropic displacement parameters (Å^2^)

**Table:**

	*x*	*y*	*z*	*U*_iso_*/*U*_eq_	
Cd1	1.01826 (6)	0.20199 (3)	0.2500	0.03793 (14)	
I1	0.64576 (5)	0.22518 (3)	0.2500	0.03959 (14)	
I2	1.14547 (4)	0.28284 (2)	0.125323 (15)	0.04579 (13)	
I3	1.11401 (5)	0.01921 (3)	0.2500	0.04403 (15)	
N1	0.6396 (7)	0.4593 (3)	−0.11682 (19)	0.0606 (12)	
H1B	0.6371	0.4870	−0.1572	0.073*	
N2	0.6522 (6)	0.3361 (3)	0.0766 (2)	0.0617 (11)	
H2A	0.5548	0.3145	0.0950	0.074*	
H2B	0.7526	0.3324	0.0996	0.074*	
C1	0.4861 (8)	0.4240 (3)	−0.0903 (2)	0.0588 (13)	
H1A	0.3794	0.4282	−0.1163	0.071*	
C2	0.4861 (6)	0.3821 (3)	−0.0254 (2)	0.0467 (10)	
H2C	0.3797	0.3583	−0.0067	0.056*	
C3	0.6476 (6)	0.3755 (3)	0.0125 (2)	0.0407 (9)	
C4	0.8047 (7)	0.4105 (3)	−0.0187 (3)	0.0547 (11)	
H4A	0.9155	0.4048	0.0043	0.066*	
C5	0.7938 (8)	0.4524 (3)	−0.0821 (3)	0.0626 (13)	
H5A	0.8977	0.4772	−0.1021	0.075*	
OW1	0.4000 (7)	0.4442 (3)	0.2500	0.0552 (11)	
HW1A	0.355 (5)	0.3924 (19)	0.2500	0.083*	
HW1B	0.514 (3)	0.439 (3)	0.2500	0.083*	

### Atomic displacement parameters (Å^2^)

**Table:**

	*U*^11^	*U*^22^	*U*^33^	*U*^12^	*U*^13^	*U*^23^
Cd1	0.0368 (3)	0.0395 (3)	0.0375 (2)	0.00051 (18)	0.000	0.000
I1	0.0341 (2)	0.0429 (2)	0.0418 (2)	0.00329 (15)	0.000	0.000
I2	0.0395 (2)	0.0539 (2)	0.04392 (19)	0.00186 (12)	0.00688 (10)	0.01025 (11)
I3	0.0430 (3)	0.0383 (2)	0.0508 (2)	0.00445 (16)	0.000	0.000
N1	0.101 (4)	0.046 (2)	0.0349 (18)	−0.001 (2)	0.0029 (19)	0.0039 (16)
N2	0.059 (3)	0.072 (3)	0.054 (2)	−0.008 (2)	−0.0031 (17)	0.022 (2)
C1	0.077 (4)	0.049 (3)	0.051 (2)	0.002 (3)	−0.018 (2)	−0.009 (2)
C2	0.048 (3)	0.044 (2)	0.048 (2)	−0.002 (2)	−0.0030 (19)	−0.0044 (18)
C3	0.047 (3)	0.033 (2)	0.042 (2)	0.0019 (17)	0.0029 (16)	0.0002 (16)
C4	0.048 (3)	0.056 (3)	0.061 (3)	0.002 (2)	0.008 (2)	0.006 (2)
C5	0.071 (4)	0.061 (3)	0.056 (3)	0.000 (3)	0.023 (3)	0.001 (2)
OW1	0.067 (3)	0.051 (3)	0.047 (2)	0.001 (2)	0.000	0.000

### Geometric parameters (Å, º)

**Table:**

Cd1—I1	2.7771 (6)	C1—C2	1.363 (6)
Cd1—I3	2.7849 (6)	C1—H1A	0.9300
Cd1—I2^i^	2.7852 (4)	C2—C3	1.394 (6)
Cd1—I2	2.7852 (4)	C2—H2C	0.9300
N1—C5	1.317 (7)	C3—C4	1.400 (6)
N1—C1	1.345 (7)	C4—C5	1.341 (7)
N1—H1B	0.8600	C4—H4A	0.9300
N2—C3	1.333 (5)	C5—H5A	0.9300
N2—H2A	0.8600	OW1—HW1A	0.83 (2)
N2—H2B	0.8600	OW1—HW1B	0.85 (2)
			
I1—Cd1—I3	111.805 (18)	C2—C1—H1A	119.8
I1—Cd1—I2^i^	106.423 (13)	C1—C2—C3	119.1 (5)
I3—Cd1—I2^i^	109.129 (13)	C1—C2—H2C	120.5
I1—Cd1—I2	106.423 (13)	C3—C2—H2C	120.5
I3—Cd1—I2	109.129 (13)	N2—C3—C2	120.8 (4)
I2^i^—Cd1—I2	113.94 (2)	N2—C3—C4	121.0 (4)
C5—N1—C1	121.2 (4)	C2—C3—C4	118.3 (4)
C5—N1—H1B	119.4	C5—C4—C3	119.3 (5)
C1—N1—H1B	119.4	C5—C4—H4A	120.3
C3—N2—H2A	120.0	C3—C4—H4A	120.3
C3—N2—H2B	120.0	N1—C5—C4	121.7 (5)
H2A—N2—H2B	120.0	N1—C5—H5A	119.1
N1—C1—C2	120.3 (5)	C4—C5—H5A	119.1
N1—C1—H1A	119.8	HW1A—OW1—HW1B	108 (3)

Symmetry code: (i) *x*, *y*, −*z*+1/2.

### Hydrogen-bond geometry (Å, º)

**Table:**

*D*—H···*A*	*D*—H	H···*A*	*D*···*A*	*D*—H···*A*
N1—H1*B*···O*W*1^ii^	0.86	2.03	2.886 (5)	173
N2—H2*A*···I2^iii^	0.86	3.12	3.938 (4)	161
N2—H2*B*···I2	0.86	3.04	3.843 (4)	157
O*W*1—H*W*1*A*···I2^iii^	0.83 (2)	3.24 (1)	3.828 (4)	130 (1)
O*W*1—H*W*1*A*···I2^iv^	0.83 (2)	3.24 (1)	3.828 (4)	130 (1)
O*W*1—H*W*1*A*···I1	0.83 (2)	3.27 (4)	3.704 (5)	116 (3)
O*W*1—H*W*1*B*···I3^v^	0.85 (2)	2.99 (2)	3.761 (5)	152 (4)
O*W*1—H*W*1*A*···I1	0.83 (2)	3.27 (4)	3.704 (5)	116 (3)

Symmetry codes: (ii) −*x*+1, −*y*+1, −*z*; (iii) *x*−1, *y*, *z*; (iv) *x*−1, *y*, −*z*+1/2; (v) −*x*+2, *y*+1/2, −*z*+1/2.
